# Comparative transcriptome analyses of maize seedling root responses to salt stress

**DOI:** 10.7717/peerj.10765

**Published:** 2021-03-02

**Authors:** Xiaoxiang Zhang, Peng Liu, Chunyan Qing, Cong Yang, Yaou Shen, Langlang Ma

**Affiliations:** 1 Maize Research Institute, Sichuan Agricultural University, Chengdu, Wenjiang, China; 2 State Key Laboratory of Crop Gene Exploration and Utilization in Southwest China, Sichuan Agricultural University, Chengdu, Wenjiang, China

**Keywords:** Maize seedling root, Salt stress, RNA sequencing, Salt tolerance, Differentially expressed genes

## Abstract

Salt stress affects crop yield by limiting growth and delaying development. In this study, we constructed 16 transcriptome libraries from maize seedling roots using two maize lines, with contrasting salt tolerance, that were exposed to salt stress for 0, 6, 18 and 36 h. In total, 6,584 differential expression genes (DEGs; 3,669 upregulated, 2,915 downregulated) were induced in the salt-sensitive line and 6,419 DEGs (3,876 upregulated, 2,543 downregulated) were induced in the salt-tolerant line. Several DEGs common to both lines were enriched in the ABA signaling pathway, which was presumed to coordinate the process of maize salt response. A total of 459 DEGs were specifically induced in the salt-tolerant line and represented candidate genes responsible for high salt-tolerance. Expression pattern analysis for these DEGs indicated that the period between 0 and 6 h was a crucial period for the rapid response of the tolerant genes under salt stress. Among these DEGs, several genes, Aux/IAA, SAUR, and CBL-interacting kinase have been reported to regulate salt tolerance. In addition, the transcription factors WRKY, bZIP and MYB acted as regulators in the salt-responsive regulatory network of maize roots. Our findings will contribute to understanding of the mechanism on salt response and provide references for functional gene revelation in plants.

## Introduction

Soil salinity stress is one of the most important abiotic stresses because it constrains global crop productivity (Ismail & Horie, 2017; Zhang et al., 2019a) by causing the retardation of plant growth and dysregulate various biological processes in plant (Fortmeier & Schubert, 2006; Fricke et al., 2004; Zhang et al., 2018). It is estimated that approximate 20% of the arable land is highly saline (Sandhu et al., 2020), and salinized land is expected to increase globally due to the increasing use of irrigation water with high salt content, in addition to poor agricultural management practices (Zhao, Jung & Schubert, 2019). To cope with soil salinity stress, plants have evolved various physiological mechanisms for enhancing tolerance and resistance to salt, such as ion compartmentalizing into vacuoles and ion exclusion from roots (Sandhu & Kaundal, 2018). The genes *SOS1*, *SOS2*, and *SOS3* play a critical role in the SOS pathway (Zhu, 2002). *SOS1* is a plasma membrane Na^+^/H^+^ transporter and presents the highest expression in root tip epidermal cells (Shi et al., 2002). *SOS2* encodes a serine/threonine protein kinase and is activated by salt-induced calcium signal (Liu et al., 2000). *SOS3* is an EF-hand calcium-binding protein that presumably senses calcium signal elicited by the salt stress (Halfter, Ishitani & Zhu, 2000; Zhu, 2002). In this pathway, the protein kinase complex SOS3-SOS2 is activated, which subsequently switch on the activity of ion transporter SOS1 by phosphorylation during salt stress (Quintero et al., 2011). Besides, under salt stress, high affinity K^+^ transporter enhances salt tolerance in plants by maintaining Na^+^/K^+^ homeostasis (Cao et al., 2019; Zhang et al., 2018; Zhang et al., 2019a).

Salt tolerance/resistance is a complex quantitative trait in plant (Liu et al., 2019). Many methods including QTL mapping (Cui et al., 2014), genome-wide association study (Zhang et al., 2018) and RNA sequencing analysis (Sharmin et al., 2020) have been used to study salt tolerance/resistance in plants. Of them, RNA sequencing analysis is an efficient approach that reveals the expression level of the genes controlling plant tolerance to abiotic stress (Sharmin et al., 2020). A large number of salt-induced differential expression genes (DEGs) that enhance salt tolerance in plants have been identified using transcriptomic analysis, and are classed into two types based on the functions of proteins they encode (Munns & Tester, 2008). One category includes genes encoding for effector-mediated functional proteins, such as superoxide dismutase (Liu et al., 2019; Rubioet al., 2009) and aquaporin (Shafi et al., 2015), which directly participated in salt stress responses. The other category includes genes regulating various kinases and transcription factors involved in signal transduction pathways (Chen et al., 2014; Deinlein et al., 2014; Munns & Tester, 2008; Tripathi et al., 2009).

Maize has the largest planting area of all crops and plays an irreplaceable role in global food security. As a crop sensitive to salinity stress (Farooq et al., 2015), maize shows significant intraspecific genetic variations in tolerance to salt (Luo et al., 2019; Zhang et al., 2018). Several genes have been implicated in salt tolerance in maize. For example, Zhang et al. (2018) and Zhang et al. (2019a) reported that ZmHKT1 (an HKT-type Na+-selective transporter) and ZmHAK4 (a membrane-localized Na+-selective transporter) enhanced salt tolerance by promoting shoot Na^+^ exclusion to maintain Na^+^/K^+^ homeostasis Given the importance of maize for global food security and the expected rise in salinity over time in many soils, understanding the mechanisms underlying salt tolerance in maize is highly important and a vital step towards reducing the effects of salinity on yield. Here, we used Illumina RNA sequencing to screen DEGs of a salt-tolerant maize line L2010-3 and a salt-sensitive line BML1234 at 0 6, 18, and 36 h under treatment with 150 mM NaCl (Na150) and identified several novel genes involved in maize responses and defenses to salt stress. Besides, we validated the DEGs specifically induced in the salt-tolerant line to help illuminate the mechanisms on salt tolerance in maize.

## Material and Methods

### Plant materials and salt treatment

Following a salt stress resistance test conducted on an association panel of 330 diverse maize inbred lines, two lines were selected for inclusion in this study: the salt-tolerant line L2010-3 and the salt-sensitive line BML1234. The seeds from both lines were surface-sterilized in 10% (v/v) H_2_O_2_ for 15 min, rinsed with distilled water and then germinated on filter paper saturated with distilled water. Uniform seedlings with two leaves were transplanted into aerated 1 × Hoagland’s nutrient solution + Na150 for the salt stress (Table S1) (Wang et al., 2003). The seedlings were then cultured in a growth room at 26 °C, a relative humidity of 70%, and a 14/10 h, day/night light cycle. At 0 h (control), 6 h (T-6), 18 h (T-18), and 36 h (T-36) under salt treatment, for each sample, the mixed roots from three seedlings of each line were individually collected as one replicate for transcriptome sequencing, with two biological replicates. The samples were then frozen in liquid nitrogen and stored at −80 °C for later use in transcriptome sequencing.

### RNA isolation and sequencing

According to the manufacturer’s instructions, total RNA of each sample was isolated using the HiPure Plant RNA Maxi Kit purchased from Magen (http://www.magentec.com.cn). RNA library construction and transcriptome sequencing were performed using the Illumina sequencing platform.

### RNA-seq Data processing and analysis

Raw sequencing reads were filtered to remove low-quality reads, adaptor-polluted reads, and reads containing Poly-N using fastp (Chen et al., 2018). Clean reads were subsequently mapped to the maize reference genome (ZmB73 RefGen_V4; obtained from http://www.gramene.org) via Hisat2. Gene expression levels were determined using Htseq software and were normalized to transcripts per million (TPM) using consumer script. Additionally, the R package DESeq was used to analyze the DEGs between each pairwise sample (Anders & Huber, 2010). Genes with a log_2_fold-change (FC) ≥ 1 in absolute value and false discovery rate (FDR) <0.05 were considered as the DEGs. Using the R package clusterProfiler (Yu et al., 2012), the DEGs were used to analyze Gene Ontology (GO) and Kyoto Encyclopedia of Genes and Genomes (KEGG) enrichment for identifying the enriched biological processes and metabolic pathways involved in maize salt tolerance.

### Quantitative real-time PCR (qRT-PCR)

To further validate the accuracy of the Illumina sequencing results, we conducted qRT-PCR on five randomly selected DEGs (*Zm00001d005841*, *Zm00001d053932*, *Zm00001d053939*, *Zm00001d048407*, and *Zm00001d026370*; the sequence of these genes were obtained from https://maizegdb.org/genome/assembly/Zm-B73-REFERENCE-GRAMENE-4.0) to obtain their relative expression levels in the collected samples. The primer pairs for qRT-PCR were designed using Primer 5.0 software and are shown in Table S2. The qRT-PCR was carried out with an Applied Biosystems 7500 Real-Time PCR System and the reaction program as follows: 2 min at 98 °C, 2 s at 98 °C, 10 s at 59 °C, 40 cycles. A thermal denaturing step was then performed for generation of the melting curves for amplification specificity verification. The maize actin1 gene was selected as the reference for normalizing the gene expression. Three technical replicates were set for all of the reactions. The 2^−^^△△^^CT^ method was used for calculation of the relative expression levels of target genes (Livak & Schmittgen, 2001).

## Results

### Phenotypic evaluation of the two maize lines

To evaluate the response to salt stress in maize, L2010-3 and BML1234 seedlings at trefoil stage were exposed to Na150 treatment for 72 h. Compared to L2010-3, the growth of BML1234 was inhibited in Na150, with the decreased plant height and biomass. Furthermore, the leaves and stems of BML1234 began to wilt after 72 h under Na150 (Fig. S1) (Wang et al., 2003). As described by Zhu, Zheng & Zhang (2011), a principal component analysis was used to calculate a salt tolerance index (STI) of both lines. The STI values were 0.56 and 0.27 for L2010-3 and BML1234, respectively. Combined, these findings indicated that L2010-3 is more tolerant to salt stress than BML1234.

### Assembly of transcriptomes and quality assessment

Clean reads were obtained by filtering the raw reads from two biological replicates of both maize lines taken at four time points post-Na150 treatment (*n* = 16 libraries in total). The number of clean reads was 125.54 million in average with a range of 118.90 to 138.09 million among the samples (Table S3). Besides, The Q20 values were all larger than 97.09%, and the Q30 values ranged from 92.35–93.84%, indicating that the sequence data were high-quality. Gene expression levels were further normalized as TPM using Cufflinks software (Table S4). Next, a correlation analysis between biological replicates was performed with the RNA-seq data. As shown in Fig. S2, high Pearson’s correlations (R^2^ ≥ 0.90) between replicates were observed, indicating that our sequencing results were highly repeatable.

### Identification of DEGs in the lines with contrasting salt tolerance

The DEGs were examined according to the criteria: —log_2_(FC)— ≥1 and FDR < 0.05. The results of seven comparisons from the eight samples per line are shown in Fig. S3 and Table S5 . Quite difference exists between the genetic backgrounds, reflected by 3,532 DEGs identified via comparing the expressions between the two lines at 0 h. Relative to the gene expression levels at 0 h, 1,800, 1,898, 2,886 genes were differentially expressed in BML1234 at 6, 18, and 36 h in salt stress, respectively. Of these, 1,122, 1,274, and 1,273 DEGs, respectively, were upregulated. There were 788 DEGs common to all of salt stress periods and 300, 294, and 428 were detected in the pairwise salt exposure comparisons T06 vs. T18, T06 vs. T36, and T18 vs. T36, respectively (Fig. S4A). Furthermore, the number of DEGs in BLM1234 between 0 h and 36 h in salt stress was much higher than others, indicating that a large number of the DEGs were involved maize stress responses after 36 h of salt exposure. In L2010-3, we detected 2,141, 1,949, and 2,329 DEGs that were responsive to salt stress at 6, 18, and 36 h, respectively. Among these, 1,320, 1,149, and 1,407 DEGs, respectively, were upregulated. In total, there were 785 DEGs and 214, 383, and 451 genes were differentially expressed in the T06 vs. T18, T06 vs. T36, and T18 vs. T36 comparisons, respectively (Fig. S4B). Principal component analysis showed that there were small differences within groups and large differences between groups, indicating that salt response- and tolerance- associated genes may be primary drivers of between-group differences (Fig. S5).

**Figure 1:**
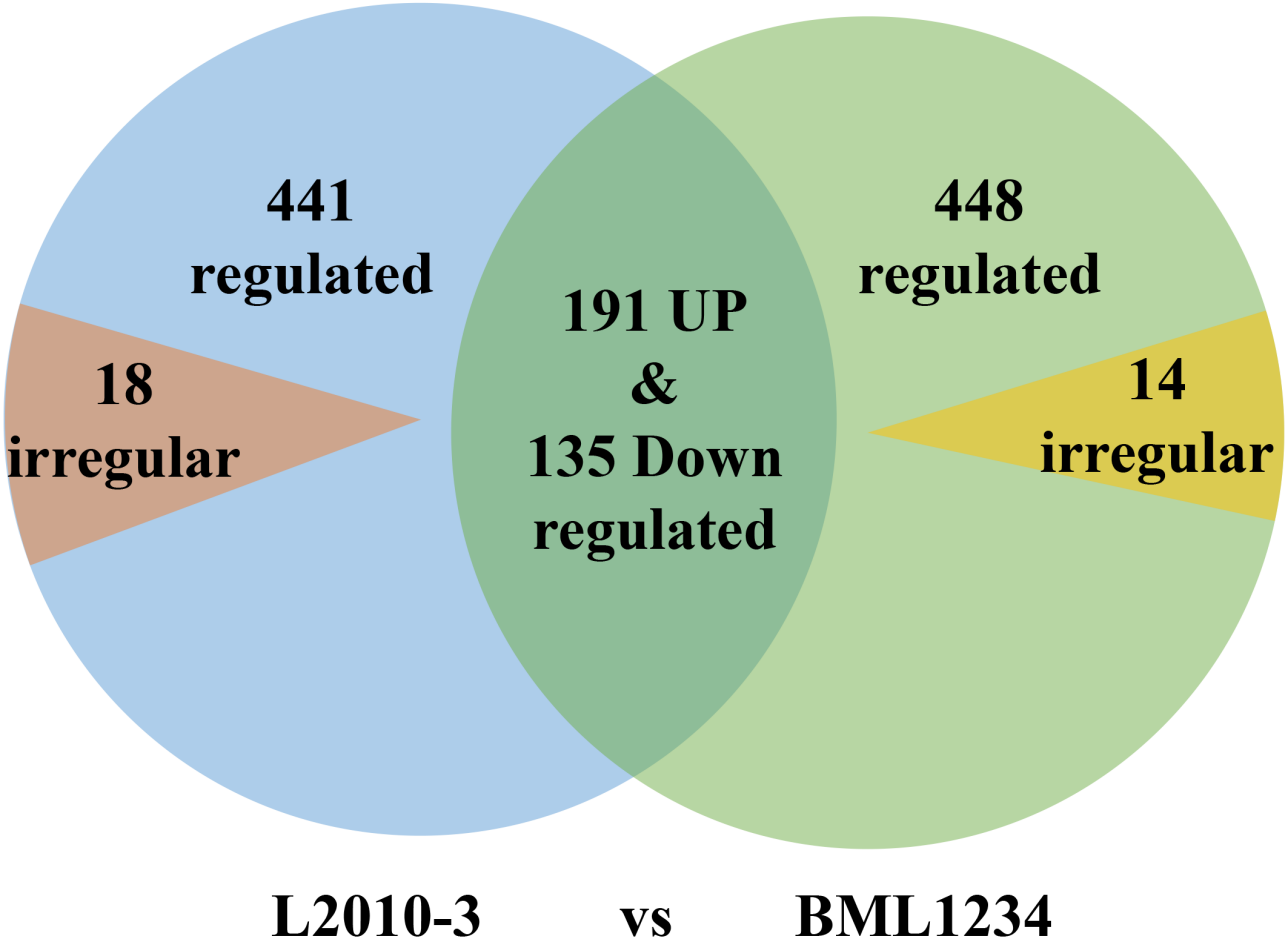
Venn diagrams of differentially expressed genes common to the two inbred lines.

More DEGs were observed at each stage of salt exposure (0–6, 6–18, and 8–36 h) in BML1234 than in L2010-3, which could partially account for the higher salt tolerance of L2010-3. We also assessed the DEGs common to all salt exposure stages t for both lines (Fig. 1, Tables S6 –S8). In total, 774 DEGs were consistently upregulated or downregulated and 14 DEGs were irregularly expressed across all salt exposure stages in BML1234. Similarly, 767 DEGs were consistently regulated and 18 were irregularly expressed across all salt exposure stages in L2010-3. There were 326 consistently regulated DEGs shared by both lines, implying common mechanisms of salt response. More importantly, the 459 DEGs that were specifically induced in the salt-tolerant line L2010-3 might represent the candidate genes involved in maize tolerance to salt stress and should be given priority for further functional revelation.

### Validation of candidate gene expression

To confirm the reliability of the RNA-seq data, we randomly selected five DEGs for qRT-PCR analysis to obtained their relative expression levels at each salt exposure stage in both lines. The results indicated that a high correlation existed between the RNA-seq data and qRT-PCR results, with the correlation coefficients ranging from 0.79 to 0.98. This verified that the identified DEGs were reliable (Fig. S6).

### Salt response- and tolerance- associated DEGs

**Figure 2:**
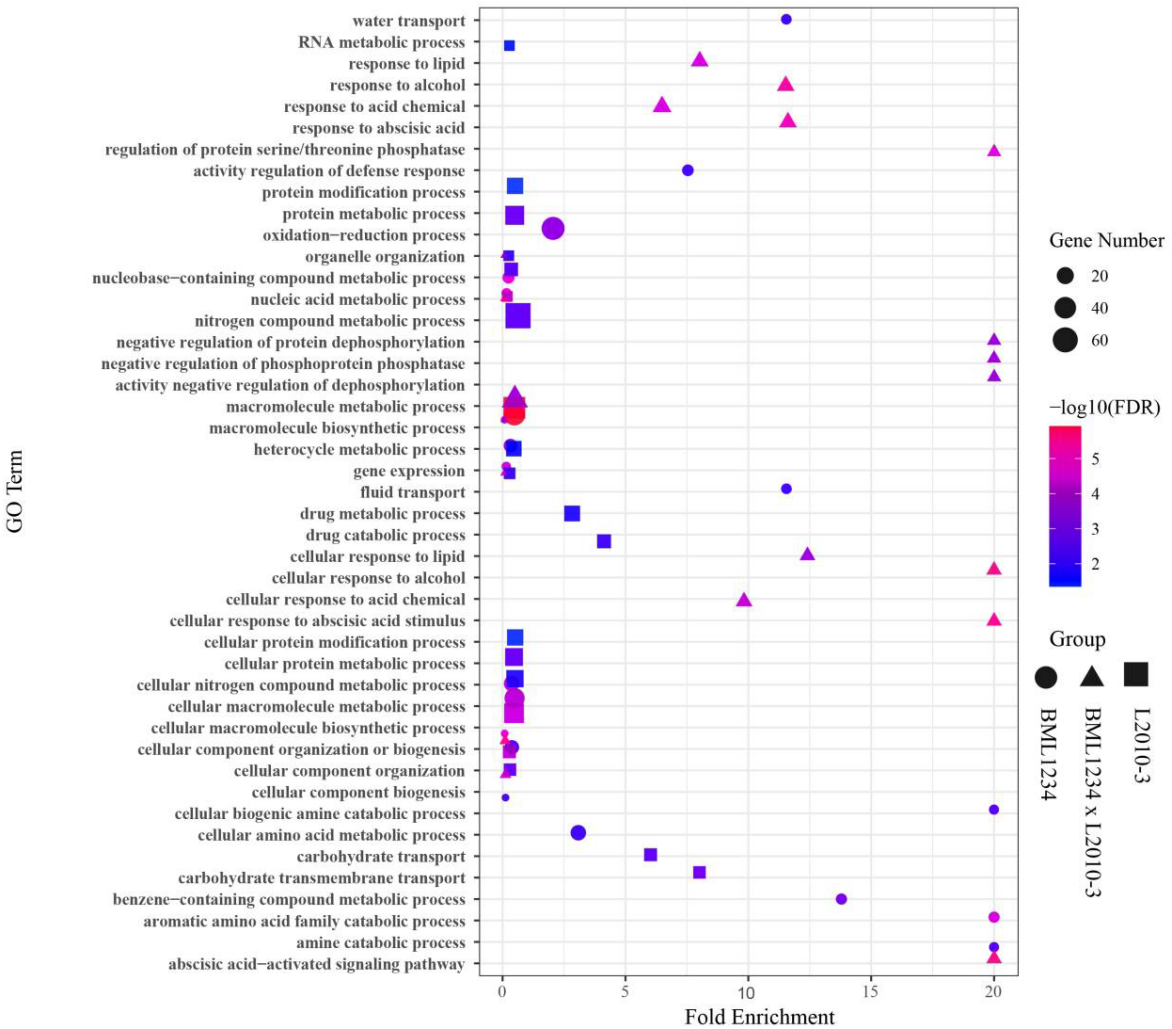
Enriched GO terms related to specifically regulated DEGs in two lines under salt stress. The ordinate represents the top 20 biological process GO terms. The abscissa represents the fold enrichment factor.

To determine if the DEGs identified in salt stress had any functional connection to salt response and tolerance, we performed GO analysis of the DEGs common to both lines and those specific to each line. Large proportions of the common DEGs were closely related to plant salt response or defense pathways (Fig. 2, Table S9), such as macromolecule metabolic process (GO:0043170), response to stress (GO:0006950). In addition, a number of common DEGs were enriched in the terms of response to abscisic acid (GO:0009737), cellular response to abscisic acid stimulus (GO:0071215), and abscisic acid-activated signaling (GO:0009738). It suggested that abscisic acid (ABA) pathways play vital roles in response to salt stress. Remarkably, some specific DEGs in L2010-3 were enriched in the term of defense response (GO:0006952), which was assumed to correlate with tolerance of salt stress in maize.

**Figure 3:**
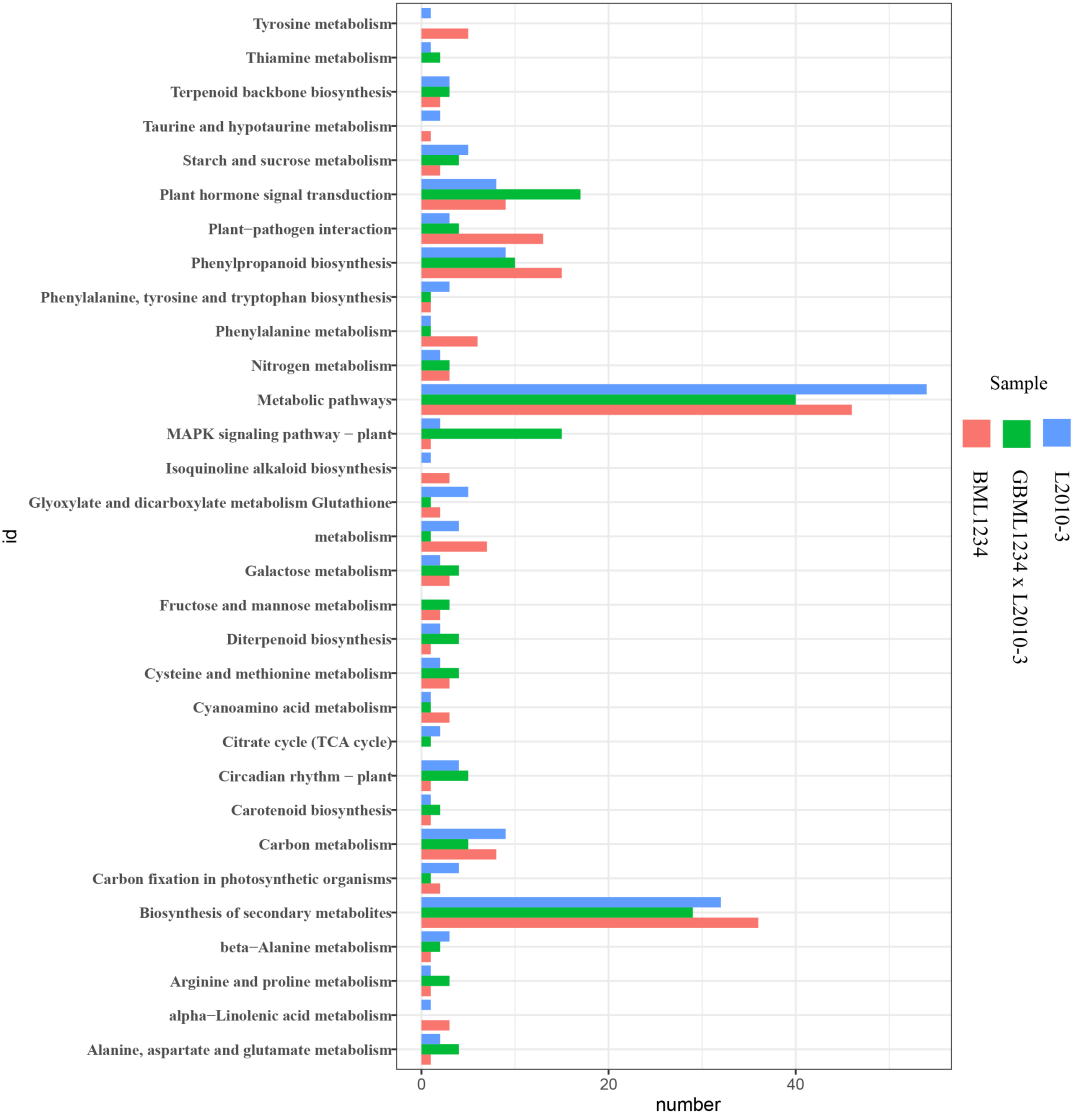
Enriched KEGG pathways related to specific DEGs in the two lines. The ordinate represents the top 20 biological pathways in KEGG. The abscissa represents the numbers of genes related to each pathway.

To further reveal the functional roles of these DEGs involved in salt response, KEGG analysis was carried out (Fig. 3, Table S10). Several KEGG pathways were significantly enriched by these DEGs, such as the metabolic pathways, biosynthesis of secondary metabolites, plant hormone signal transduction, and phenylpropanoid biosynthesis. Of these, the metabolic pathways and biosynthesis of secondary metabolites were the most significant pathways.

### Expression patterns of specific DEGs in the salt-tolerant line

**Figure 4:**
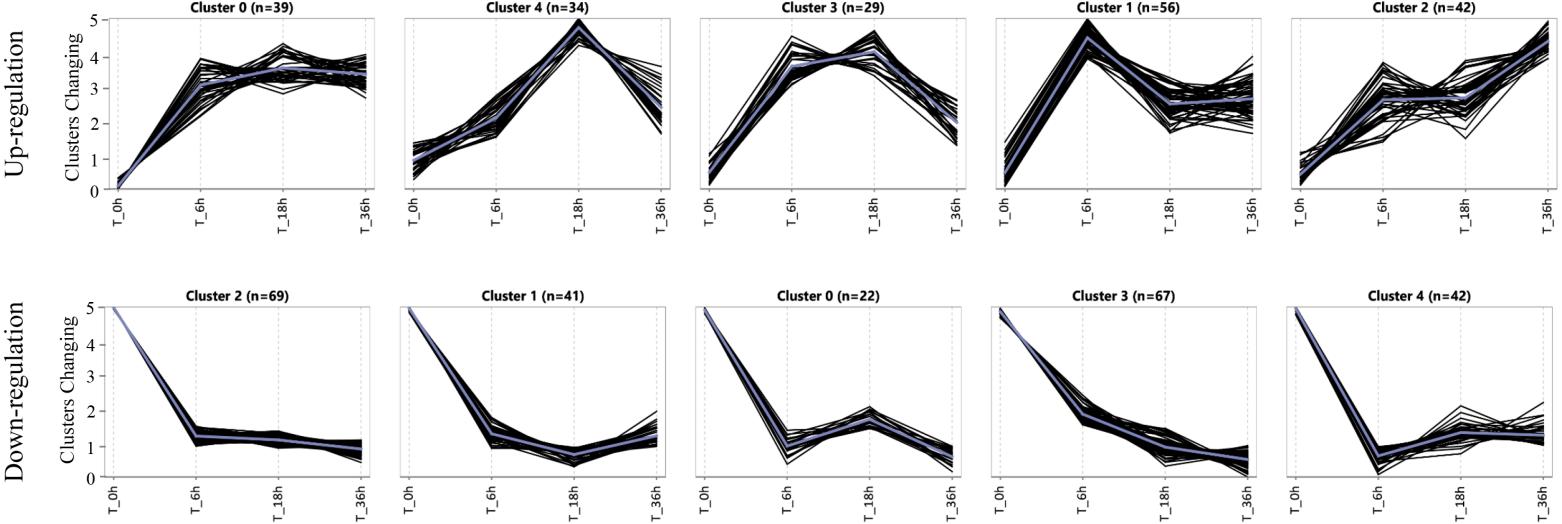
The expression patterns of specific genes in L2010-3.

To assess the expression patterns of the salt tolerance-related genes, we carried out a K-means approach for the specific DEGs induced in the tolerant line L2010-3 by using ExpressCluster software (Zhang et al., 2019b). As shown in Fig. 4 and Table S8, these upregulated and downregulated specific DEGs were grouped into five clusters according to their expression patterns. Interestingly, all the clusters showed the most dramatic changes in DEGs at stage I, indicating that stage I was a crucial period for the rapid response of the tolerant genes under Na150. The up-regulated genes in Cluster 1 were increasingly expressed at between 0 and 6 h of salt exposure, and returned to a lower expression levels during the subsequent stages. These genes appear to be instantaneously responding class and thus may be only required for early response to Na150 stress. The upregulated genes in Cluster 2 and the downregulated genes in Cluster 1 displayed to continuously increase and decrease, respectively, throughout all the stages. These genes are likely key in regulating salt tolerance in maize and several have been shown previously to respond to salt stress, such as *Zm00001d023516* (salt stress-induced protein) and *Zm00001d044642* (CBL-interacting kinase).

### Characteristics of genes associated with salt tolerance

In this study, the DEGs specifically induced in the tolerant line was taken as the genes associated with salt tolerance in maize. Among the specific DEGs common to three stages in L20110-3, numerous DEGs were involved in hormonal regulations, ion homeostasis and antioxidant mechanism.

Hormonal regulations: Previous reports have shown the important roles of hormones in plant tolerance to salt stress (Deinlein et al., 2014; Farooq et al., 2015). In L20110-3, a total of seven (six upregulated and one downregulated) specific DEGs were involved in plant hormonal regulation (Table S8). Among the six upregulated genes, *Zm00001d018414*, *Zm00001d017284*, and *Zm00001d018973* encode Aux/IAA-transcription factors, whereas *Zm00001d002374* was annotated to be a SAUR-like auxin-responsive protein. Additionally, *Zm00001d017612* encoded brassinosteroid responsive RING-H2 and *Zm00001d051584* was annotated as ethylene response protein. The one downregulated gene *Zm00001d002999* encodes gibberellin 2-oxidase 2. In previous studies, the orthologues of these genes were shown to regulate salt tolerance in *Arabidopsis*, tobacco and wheat (Hu et al., 2015; Jin et al., 2016; Liu & Zhu, 1997).

Ion homeostasis: Maintenance of the stable K^+^/Na^+^ could increase the tolerance to salt stress; several proteins, including serine/threonine protein kinases, CBL-interacting kinase, and Ca^2+^-regulated proteins, were identified as being associated with K^+^/Na^+^ balance by previous studies (Farooq et al., 2015; Xie et al., 2018; Zhu, 2003). Among the DEGs specific to L20110-3, five genes were involved in maintaining ion homeostasis in maize under salt stress (Table S8).

Antioxidant mechanism: Under salt stress, reactive oxygen species were over-generated and then result in oxidative stress in plants (Farooq et al., 2015). However, plants evolved antioxidant defense mechanisms against oxidative stress. Many genes, including peroxidase, thioredoxin, glutaredoxins and S-transferase, exist in antioxidant defense pathway (Luan et al., 2020; Xie et al., 2018). In the specific DEGs to L2010-3, we identified 14 genes involved in antioxidant mechanism (Table S8), of which three genes (*Zm00001d018238*, *Zm00001d041804* and *Zm00001d018461*) encode thioredoxin. Besides, respectively eight and two genes were annotated as peroxidase and glutathione S-transferase.

Other defense mechanisms: Among the specific DEGs of L2010-3, we also identified several genes involved in stress defense, including leucine-rich repeat protein (*Zm00001d035756*) and pathogenesis-related protein (*Zm00001d018324*). Of these, leucine-rich repeat protein was directly involved in cell wall loosening causing root growth (Kwon et al., 2015). Pathogenesis-related protein were reported to directly participate in response to salt stress (Seo et al., 2008).

Transcription factors (TFs) are a class of regulators that participate in plant growth, development and stress responses. Using the PlantTFDB database (http://planttfdb.gao-lab.org/), we identified 108 TFs belonging to 24 families (Table S11). Of these, 35 TFs distributed among 21 families were specifically upregulated or downregulated in L2010-3 under Na150. The genes *Zm00001d053220*, *Zm00001d005622*, and *Zm00001d043992* belong to MYB, WRKY and bZIP TF families, respectively, which were previously reported to activate the tolerant capability under salt treatment (Xie et al., 2018). More importantly, these genes were among the top 10 DEGs (Tables S6–S8, verifying their important roles in salt tolerance.

According to the GO and KEGG analysis (Tables S9, S10), *Zm00001d024210* (terpene synthase) and *Zm00001d032858* (kaurene synthase) were significantly enriched in defense response (GO:0006952) and metabolic pathways, which are extensively considered strongly related to plant salt resistance (Basyuni et al. 2009; Xie et al., 2018; Wang et al., 2019). Among the specific DEGs in L20110-3, some genes that encode peroxidases were continuously downregulated throughout the stages of salt stress (Table S8). The downregulation of peroxidase genes may be deemed an important indicator of plant defense against high salinity (Wang et al., 2019).

## Discussion

### ABA signal pathway coordinates the response to salt stress in maize

Tolerance and response to salt stress is a complex procedure in plants. After perception of salt stress, a complex network is activated and culminates in resistance to salt toxicity, including the hormone (e.g., ABA; cytokinin) signaling transduction, antioxidant defense, osmotic adjustment, and regulations in defense gene (Farooq et al., 2015; Fricke et al., 2004; Long, Zou & Zhang, 2015; Lu et al., 2019; Wu et al., 2019). In particular, ABA signaling transduction is a critical pathway in response to salt stress. The DEG overlaps between BML1234 and L2010-3 represent these genes in the common pathways of response to salt stress in maize. In this study, quite a few DEGs common to the both lines were enriched in ABA signaling pathway, such as abscisic acid receptor (*Zm00001d038436*, *Zm00001d016294*, and *Zm00001d028793*) and abscisic acid-insensitive 5-like protein (*Zm00001d018178*). They probably acted as the signal regulators, which coordinated the process of salt response. Among them, *Zm00001d018178* was significantly enriched in the terms “response to salt stress” (GO:0009651) and “abscisic acid-activated signaling pathway” (GO:0009738). These observations corroborate with previous studies that found a close relationship between the ABA signaling pathway and plant responses to salt stress (Jin et al., 2016; Wang et al., 2019).

### Specific DEGs in L2010-3 correlate with the tolerance

Based on the functional annotation and expression analysis, we found that numerous genes in L2010-3, including TFs, hormonal regulations, ion homeostasis, antioxidant, participated in maize tolerance to salt stress. Likewise, these observational findings were found in previous studies and these hormones are critical in in plant tolerance to salinity (Deinlein et al., 2014; Farooq et al., 2015; Liu et al., 2019). Besides, in this study, several peroxidases were among the specific DEGs that were induced in the tolerant line. A previous study reported that the downregulation of peroxidases may promote the elongation of root by reducing apoplastic H_2_O_2_ and generating oxygen radicals (Hu et al., 2015). Moreover, some DEGs were continuously upregulated or downregulated across all three salt exposure stages and may be key genes controlling salt tolerance. Among these, CBL-interacting kinase was previously demonstrated to reduce the accumulation of Na^+^ under salt treatment (Chen et al., 2014; Tripathi et al., 2009). Salt stress-induced protein, the pathogenesis-related protein and the leucine-rich repeat protein were uncovered to involve salt stress response in plant (Guo et al., 2018; Jain et al., 2011; Liu & Zhu, 1997). These observations were supported by previous studies (Wang et al., 2019; Xie et al., 2018).

The KEGG analysis indicated that biosynthesis of secondary metabolites and metabolic pathway were significantly enriched with the specific DEGs. It suggested that these two pathways exert an essential effect on repressing or activating the resistance to salt stress in maize, which was consistent with the previous report (Wang et al., 2019). In total, these results provide a detailed explanation of the mechanism behind the high salt tolerance of L2010-3.

### Transcription factors (TF) involved in response to salt stress

Transcription factors, including NAC, MYB, AP2/EREBP and HSF, play vital roles in salt tolerance (Hu et al., 2015; Xie et al., 2018). The expressions of these TFs significantly enhanced the salt tolerance of transgenic plants (Deinlein et al., 2014). The gene *ERF76* that encodes salt-responsive ERF TF in poplar was overexpressed in transgenic tobacco and resulted in improved salt tolerance (Yao et al., 2016). Similarly, salt tolerance in tobacco was increased by overexpression of *VvIAA18* from grapevine (Li et al., 2020). Xie et al. (2018) reported that 75 TFs distributed among 10 families (NAC, AP2/ERF, GATA, MYB, bZIP, bHLH, CBF, ZFP, WRKY and SPL TF) were differentially expressed in maize under salt stress (Xie et al., 2018). In this study, among the all the DEGs, 108 were annotated as TFs (Table S11) with 33 down-regulated and 75 up-regulated. Among them, several TFs (WRKY, bZIP, bHLH and MYB) that were induced by salt stress were previously reported involved in drought and aluminum stresses (Li et al., 2017a; Li et al., 2017b), implying that these TFs were generally involved in the response of multiple stresses. Two Aux/IAA-transcription factors were specifically regulated in L2010-3, which may contribute to the high salt-tolerance of L2010-3. Interestingly, except bZIP, bHLH, ZFP, MYB-related and AP2/EREBP families, the other TF families were all up-regulated under Na150, implicating their positive roles in salt resistance. Similar phenomenons were also reported previously (Li et al., 2017c; Xie et al., 2018). These TFs monitor irregular signals under salt stress condition and accordingly regulate the expressions of the downstream genes in a negative or positive manner (Sun et al., 2015). On the whole, our results show the central roles that TFs play in the salt-responsive regulatory network in maize roots.

## Conclusions

By comparing the RNA-seq data of maize seedlings in salt-tolerant and salt-sensitive lines during exposure to salt stress, the common and specific genes were separated. Analysis of the DEGs common to both lines revealed that the ABA signaling pathway likely coordinates the process of maize salt response. Additionally, analysis of the specific genes to L2010-3 suggested that the salt-tolerant line exhibited specific functional genes involved in salt tolerance, including Aux/IAA, SAUR, and CBL-interacting kinase. Our findings help to illuminate the mechanism behind salt responses and provide reference data for functional gene revelation in plants in the future.

##  Supplemental Information

10.7717/peerj.10765/supp-1 Supplemental Information 1 Supplemental Figures. **Figure. S1** Phenotypes in maize lines L2010-3 and BML1234 at 72 h under 150 mM NaCl treatment. (A) the salt-tolerant line L2010-3. (B) the salt-sensitive BML1234. **Figure. S2** Spearman’s correlation analysis between biological replicates for RNA-seq at three stages. Color levels correspond to the degree of correlation in each replicate. **Figure. S3** Numbers of DEGs in the salt-tolerant line (L2010-3) and salt-sensitive line (BML1234) under normal condition (0 h) and salt treatment (from 6 to 36 h). **Figure. S4** Venn diagrams of genes differentially expressed at the three stages in the salt-sensitive line BML1234 and the salt-tolerant line L2010-3. **Figure. S5** Correlation analysis between RNA-seq and qPCR data.

10.7717/peerj.10765/supp-2 Supplemental Information 2 The detailed composition of the full-strength nutrient solution

10.7717/peerj.10765/supp-3 Supplemental Information 3 Primers of real-time qRT-PCR assay used for DEGs in this study

10.7717/peerj.10765/supp-4 Supplemental Information 4 Summary of clean reads identified in this study

10.7717/peerj.10765/supp-5 Supplemental Information 5 TPM values normalized in this study

10.7717/peerj.10765/supp-6 Supplemental Information 6 The DEGs in the salt-tolerant line (L2010-3) and salt-sensitive line (BML1234)

10.7717/peerj.10765/supp-7 Supplemental Information 7 Expression of differential genes common to the lines at all the stages

10.7717/peerj.10765/supp-8 Supplemental Information 8 Differential gene expression across the three stages in BML1234

10.7717/peerj.10765/supp-9 Supplemental Information 9 Differential gene expression across the three stages in L2010-3

10.7717/peerj.10765/supp-10 Supplemental Information 10 List of GO terms (BP) for the 326 DEGs common to the both lines and the DEGs unique to each line

10.7717/peerj.10765/supp-11 Supplemental Information 11 List of KEGG pathways for the 326 DEGs common to the both lines and the DEGs unique to each line

10.7717/peerj.10765/supp-12 Supplemental Information 12 Transcription factors involved in salt stress

## Additional Information and Declarations

### DNA Deposition

The following information was supplied regarding the deposition of DNA sequences: The raw sequence data are available in the NCBI Sequence Read Archive: PRJNA647980.

### Data Availability

The following information was supplied regarding data availability: The raw gene expression data are available in the Supplementary Files Table S4 and at Figshare. Raw RT-PCR data is also available at the same Figshare repository: Zhang; Xiaoxiang; Liu, Peng; Qing, Chunyan; Yang, Cong; Shen, Yaou; Ma, Langlang (2020): PeerJ-RT-PCR.xlsx. figshare. Dataset. https://doi.org/10.6084/m9.figshare.12640007.v1. Raw sequencing data are available at NCBI SRA: SAMN15615587, SAMN15615586, SAMN15615585, SAMN15615584, SAMN15615583, SAMN15615582, SAMN15615581, SAMN15615580, SAMN15615579, SAMN15615578, SAMN15615577, SAMN15615576, SAMN15615575, SAMN15615574, SAMN15615573, SAMN15615572.
