# Supplementary material for: Comparative transcriptome analyses of maize seedling root responses to salt stress
Source: PeerJ. 2021 Mar 2;9:e10765. doi: 10.7717/peerj.10765 (PMC7934676; doi:10.7717/peerj.10765)
Supplement: Supplemental Information 1 — Figure. S1 Phenotypes in maize lines L2010-3 and BML1234 at 72 h under 150 mM NaCl treatment. (A) the salt-tolerant line L2010-3. (B) the salt-sensitive BML1234. Figure. S2 Spearman’s correlation analysis between biological replicates for RNA-seq at three stages. Color levels correspond to the degree of correlation in each replicate. Figure. S3 Numbers of DEGs in the salt-tolerant line (L2010-3) and salt-sensitive line (BML1234) under normal condition (0 h) and salt treatment (from 6 to 36 h). Figure. S4 Venn diagrams of genes differentially expressed at the three stages in the salt-sensitive line BML1234 and the salt-tolerant line L2010-3. Figure. S5 Correlation analysis between RNA-seq and qPCR data. [file peerj-09-10765-s001.pdf]

Supporting information

Additional supporting information may be found in the online version of this article:

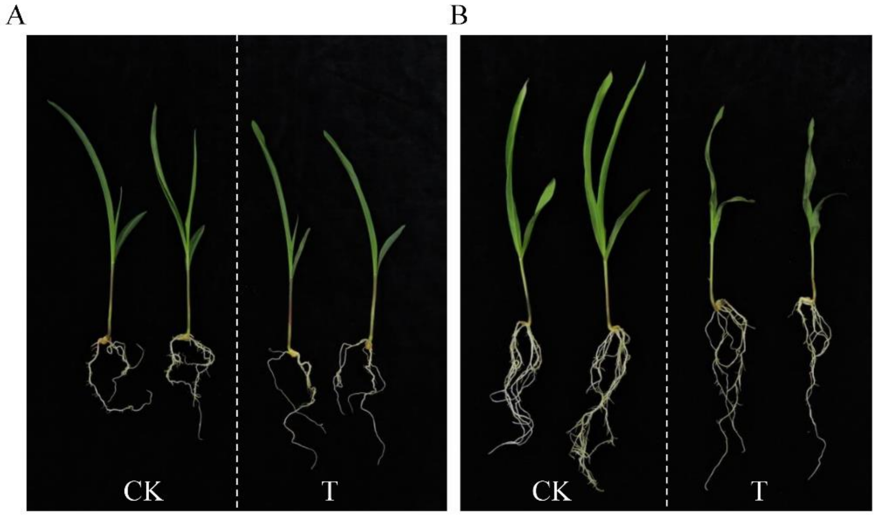

Figure. S1 Phenotypes in maize lines L2010-3 and BML1234 at 72 hours under 150 mM NaCl treatment. (A) the salt-tolerant line L2010-3. (B) the salt-sensitive BML1234.

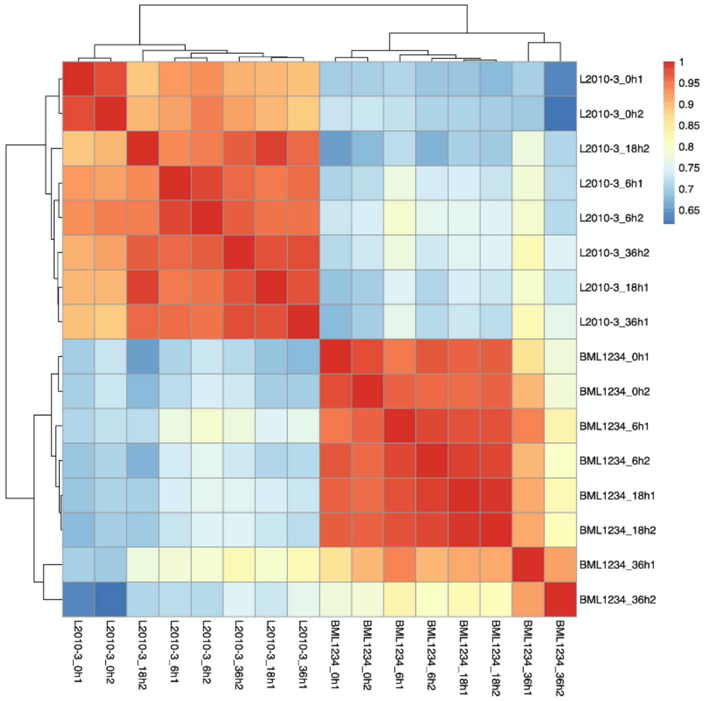

Figure. S2 Spearman's correlation analysis between biological replicates for RNA-seq at three stages. Color levels correspond to the degree of correlation in each replicate.

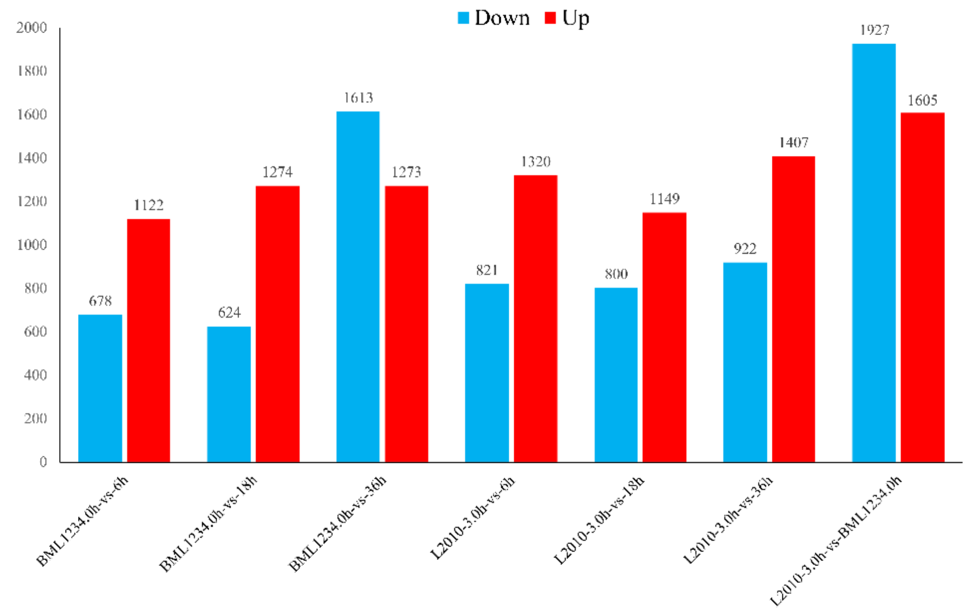

Figure. S3 Numbers of DEGs in the salt-tolerant line (L2010-3) and salt-sensitive line (BML1234) under normal condition (0 h) and salt treatment (from 6 to 36 h).

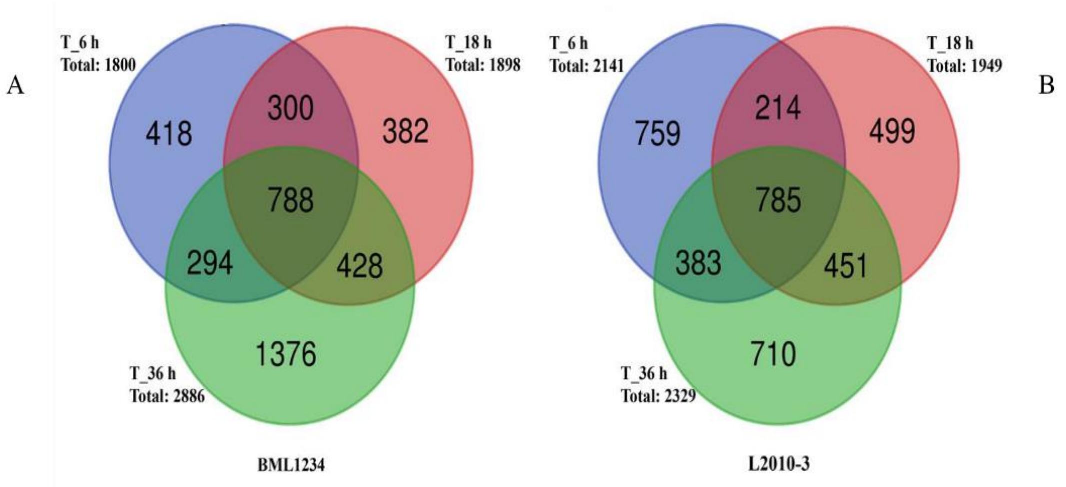

Figure. S4 Venn diagrams of genes differentially expressed at the three stages in the salt-sensitive line BML1234 and the salt-tolerant line L2010-3.

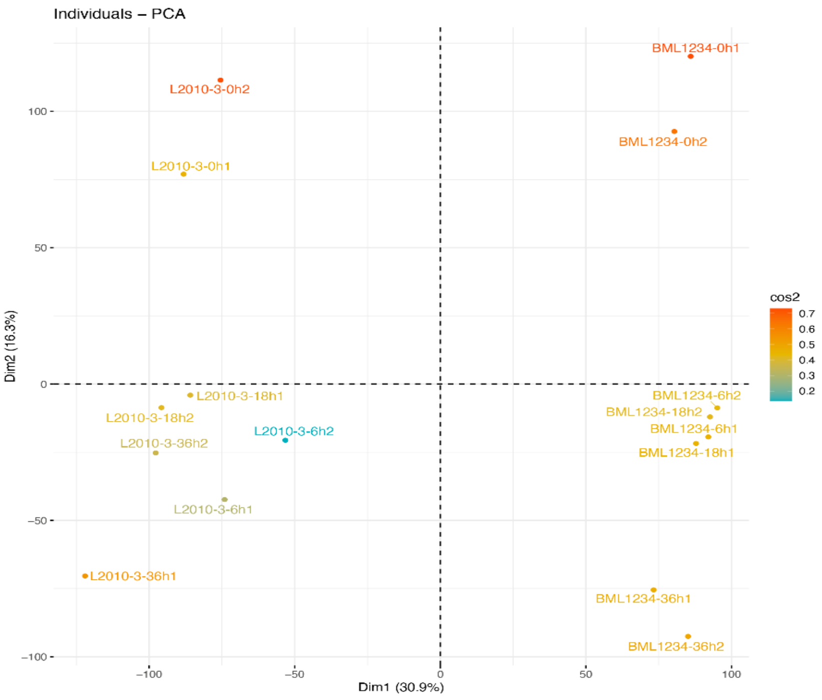

Figure. S5 Principal Component Analysis (PCA) diagram for the 16 libraries.

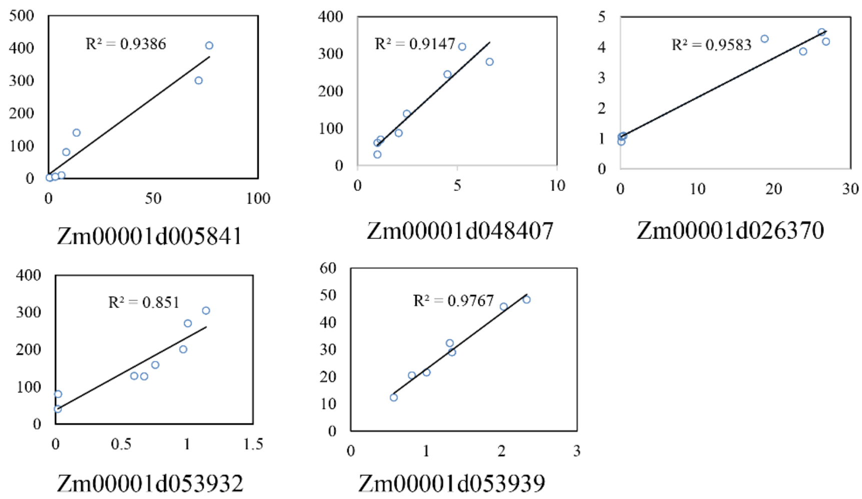

Figure. S6 Correlation analysis between RNA-seq and qPCR data.
